# Advanced enhancement technique for infrared images of wind turbine blades utilizing adaptive difference multi-scale top-hat transformation

**DOI:** 10.1038/s41598-024-66423-0

**Published:** 2024-07-06

**Authors:** Yinchao He, Shuang Kang, Wenwen Li, Hongyan Xu, Sen Liu

**Affiliations:** 1 School of Information and Control Engineering, Jilin Institute of Chemical Technology, Jilin, 132022 China; 2 School of Mechanical and Control Engineering, BaiCheng Normal University, BaiCheng, 137000 China; 3 College of Food Science and Technology, Henan Key Laboratory of Cereal and Oil Food Safety Inspection and Control, Henan University of Technology, Zhengzhou, 450001 China

**Keywords:** Engineering, Electrical and electronic engineering, Energy infrastructure, Mechanical engineering

## Abstract

Enhancing infrared images is essential for detecting wind turbine blades using infrared technology. This paper introduces an Infrared Image Enhancement Method based on Adaptive Iterative Cutoff Threshold Difference Multi-Scale Top-Hat Transformation (AICT-DMTH) to address the challenge of low image clarity in infrared detection. The method involves performing a black-white difference top-hat transformation by utilizing structural elements of varying scales for dilation and erosion. Additionally, an iterative threshold method is applied to extract more detailed image features, followed by setting a cutoff constant to determine the final scale of the structural element. The effectiveness of the proposed method is evaluated both qualitatively and quantitatively, with infrared images from laboratory and wind farm settings enhanced and compared against existing methods. The experimental results indicate that the proposed method significantly improves the clarity of infrared images, demonstrating robustness in enhancing images from various environments.

## Introduction

In recent years, there has been a steady increase in the number of wind turbines. The typical service life of wind turbine blades is approximately 20 years^1^. When a blade breaks, it not only leads to financial losses but also poses a safety risk to nearby facilities and personnel. Currently, there are limited detection methods available for wind turbine blades, with common non-destructive testing techniques including acoustic emission testing, ultrasonic testing, and infrared thermal wave testing^2–4^. This study introduces a design of an infrared thermal wave detection algorithm specifically tailored for wind turbine blades. However, two challenges were encountered during the image acquisition process: (1) Wind turbine blades have a non-planar structure with a certain curvature, resulting in uneven illumination in the infrared images; (2) The obtained infrared images under active thermal excitation exhibit low clarity and blurred details. These issues significantly impact the accurate assessment of the interior of wind turbine blades. To tackle this, a method for enhancing wind turbine blade infrared images based on mathematical morphology is proposed. Initially, a novel morphological enhancement operator is derived using morphological top-hat operation considering the difference in structural element scales. Subsequently, more scale details are obtained by adaptive iterative thresholding. Lastly, the images are fused with difference scale weighting to obtain a new enhanced image. To validate the efficacy of the proposed method, it is compared against current state-of-the-art image enhancement techniques and subjected to both qualitative and quantitative analyses. The experimental results confirm that the algorithm performs excellently across various test image sets, significantly enhancing the edge features and contrast of defects in the images. The algorithm effectively addresses issues such as uneven illumination and low image clarity caused by non-planar structures, holding significant value and broad application prospects for the non-destructive testing of wind turbine blades.

The paper provides a comprehensive overview of existing image enhancement methods and discusses their limitations in Section "Related work". In Section "AICT-DMTH method", an adaptive iterative cutoff threshold difference multiscale top-hat transform method for enhancing infrared images is presented. The experimental setup and results for infrared images are detailed in Section "Experiments". The paper concludes with a discussion of the experimental results and outlines the future implications of this research.

## Related work

### Frequency domain-based enhancement method

Frequency domain enhancement is rooted in wavelet transform, which employs various wave filters to process the frequency domain image. The resulting filtered image is then reconstructed through inverse transformation, leading to image enhancement. Common techniques include Discrete Fourier Transform (DFT), Discrete Wavelet Transform (DWT), Discrete Cosine Transform (DCT), among others. In recent years, researchers have integrated wavelet transform with other image processing methodologies to enhance image processing capabilities. For instance, a method of image fusion utilizing multi-scale Gaussian filtering and morphological transformation was proposed based on wavelet transform^5^. Additionally, defect detection was enhanced through an improved multi-scale wavelet transform approach^6^, an image enhancement algorithm was developed by combining weighted guided filtering and wavelet transform^7^, and a wavelet transform-based image enhancement method leveraging contrast entropy was introduced^8^. Nonetheless, a common limitation of these approaches lies in the complexity of parameter tuning.

### Image enhancement method based on histogram equalization

Histogram equalization (HE)^9^ is a technique that enhances contrast and improves image quality. However, the HE algorithm may introduce uneven brightness in the processed image. To mitigate this issue, researchers have proposed alternative methods such as bidirectional histogram equalization^10^ and a binary sub-image algorithm based on 2D histogram analysis^11^. Adaptive histogram equalization (AHE)^12^ is effective for enhancing local contrast but may introduce excessive noise in uniform regions. Contrast Limited Adaptive Histogram Equalization (CLAHE)^13^ addresses some of the limitations of the AHE algorithm. Recently, Multi-scale Adaptive Bi-histogram Equalization (MABHE)^14^ has demonstrated promising results in image enhancement. Nevertheless, image enhancement techniques based on histogram equalization struggle to adapt to complex image processing tasks, which remains a significant drawback of this approach.

### Application of multiscale methods in the field of images

Mathematical morphology has found widespread application in image processing, effectively enhancing image details in scenarios characterized by uneven illumination. The structural element (SE) has undergone evolution from its initial form as a line segment to encompass various shapes such as square, diamond, and circle. Calculations have progressed from single-directional to multi-directional, and scales have expanded from single-scale to multi-scale^15–17^. Hassanpur et al. introduced a multi-scale morphological top-hat transform image enhancement technique^18^, with the Contrast Improvement Ratio (CIR) serving as the objective function. However, the method may lead to local over-enhancement as the selected structural elements increase, representing a primary limitation. To address the scale selection challenge of structural elements in Hassanpur's approach, Bustacara et al. proposed an automatic stop criterion for contrast enhancement based on multi-scale top-hat transform^19^. Chen et al. utilized multi-scale top-hat transform for enhancing the contrast of infrared images^20^, while Lu et al. combined multi-scale top-hat transform with Gabor and match filters^21^. A notable drawback of this method is the lengthy computational process. César et al. presented a top-hat transform image enhancement algorithm incorporating multi-scale geodesic reconstruction (MGRTH)^22^, which significantly enhances the overall gray level, potentially leading to insufficient contrast. Building upon the MGRTH method, Mello et al. refined the image enhancement operator and introduced a novel image enhancement algorithm based on geodesic reconstruction multi-scale top-hat transform (MSTHGR)^23^.

## AICT-DMTH method

This chapter primarily presents the fundamental theories and methods of morphology, the drawbacks of traditional morphological top-hat operations, the novel difference-scale morphological operators, and the adaptive iterative cutoff threshold method.

### Basic theory of morphology

Mathematical morphology is a scientific technique utilized in image processing, grounded in set theory. At its core, this method involves manipulating images using structural elements to analyze and process structural information contained within them. In mathematical morphology, dilation and erosion serve as two fundamental operations. Dilation works by expanding bright regions through the movement of the structural element, capturing the maximum value in the neighborhood at each position. Conversely, erosion, the counterpart of dilation, diminishes bright areas by selecting the minimum value in the neighborhood at each position. If S represents the structuring element and *f* denotes the grayscale image, the definitions of erosion and dilation can be expressed as follows^24^:

1 $$\begin{array}{c}\left(f\Theta S\right)\left(x,y\right)=\underset{\alpha ,\beta }{min}\left(f\left(x+\alpha ,y+\beta \right)-S\left(\alpha ,\beta \right)\right)\end{array}$$

2 $$\begin{array}{c}\left(f\oplus S\right)\left(x,y\right)=\underset{\alpha ,\beta }{max}\left(f\left(x-\alpha ,y-\beta \right)+S\left(\alpha ,\beta \right)\right)\end{array}$$

where $\Theta ,\oplus$ denote the erosion and expansion operations, respectively. (x,y) is used to represent the spatial coordinates of pixels in the original grayscale image. The spatial coordinates of the structuring element S are represented by (α, β).

Erosion operation is first applied to the image using a structuring element in opening operation, followed by a dilation operation based on the erosion result. In contrast, in closing operation, dilation operation is first applied to the image, followed by an erosion operation on the dilated image. The definitions of these two operations are given as Ref.^24^:

3 $$\begin{array}{c}\left(f\circ S\right)=\left(f\Theta S\right)\oplus S\end{array}$$

4 $$\begin{array}{c}\left(f\cdot S\right)=\left(f\oplus S\right)\Theta S\end{array}$$

The white top-hat transform is defined as the difference between the original grayscale image and the result of an opening operation applied to that image. The black top-hat transform is the difference between the result of a closing operation and the original grayscale image. These two transformations are defined as follows^24^:

5 $$\begin{array}{c}{f}_{t}=f-\left(f\circ S\right)\end{array}$$

6 $$\begin{array}{c}{f}_{b}=\left(f\cdot S\right)-f\end{array}$$

### Differential multi-Scale morphological top-hat operator

**Figure 1:**
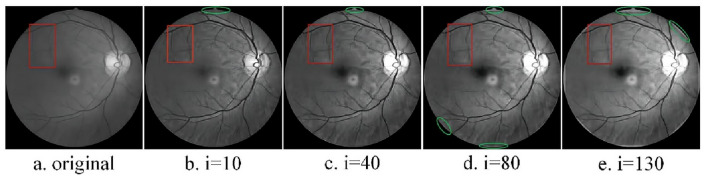
Conventional morphology multi-scale top-hat transformations.

To comprehensively assess the suitability of structural elements for image feature extraction, minimize computational complexity, and mitigate artifacts associated with morphological processing, the author opts for a circular structural element as the target of operation. The opening operation effectively eliminates bright details smaller than the structural element, with the white top-hat transform facilitating the extraction of corresponding bright features. Conversely, the closing operation filters out dark details smaller than the structural element, and the black top-hat transform aids in extracting the corresponding dark features. By performing a differential operation on the black and white top-hat transforms, detailed feature information of the image can be obtained. The expression for the image enhancement operator of the morphological top-hat transform is as follows:

7 $$\begin{array}{c}{f}_{e}=f+\left(f-f\circ S\right)-\left(f\cdot S-f\right)\end{array}$$

where ${f}_{e}$ is the enhanced image, *f* is the original grayscale image, and S is the structural element. In this paper, in order to delve into this issue, the traditional morphological top-hat transform is applied to two different objects: one is the image of the eyeball^25^, and the other is the infrared image of the crease on the blade of a wind turbine. For Fig. 1a, the scales of the selected structural elements are 10, 40, 80, and 130 respectively. The processed images are shown in Fig. 1b–e. For Fig. 3a, the scales of the selected structural elements are 3, 9, 15, and 30 respectively. The processed images are shown in Fig. 3b–e.

As illustrated in Fig. 1a–e, the enhancement of image details (highlighted by red rectangular markers) becomes more pronounced with increasing structural element scales. However, a localized issue of excessive enhancement (indicated by elliptical green markers) gradually emerges along the edge of the highlighted eyeball, impacting the overall image enhancement effect. It can be seen from Fig. 3a–e that due to the small scale of the structural elements, there is no local over-enhancement phenomenon, but as the scale of the structural elements increases, the phenomenon of local over-enhancement appears in the image (red rectangular mark). Based on the above problems, in order to enhance the details while maintaining the stability of the image. This paper proposes a new image enhancement operator. Taking the expansion operation as an example, S_2_ is obtained by expanding the structural element S_0_ by the structural element S_1_, that is ${S}_{2}={S}_{0}\oplus {S}_{1}$.

When ${S}_{i}={S}_{i-1}\oplus {S}_{1}$, i times dilation is carried out as follows:

8 $$\begin{array}{c}{S}_{k}=\underset{i}{\underbrace{S\oplus S\oplus S\cdots S\oplus S\oplus S} }\,\,\,\,\,i\ge 2\end{array}$$

Equation (9) is (i + k) times dilation:

9 $${S}_{i+k}= {\underbrace {{S \oplus S \oplus S \cdots S \oplus S \oplus S}}_{i}} {\underbrace {{ \cdots \oplus S}}_{k}}$$

According to Eqs. (3)–(6), the following formula is obtained:

10 $${f}_{t\left(i+k\right)}=f-\left(f\Theta {S}_{i}\right)\oplus {S}_{i+k}$$

11 $${f}_{b\left(i+k\right)}=\left(f\oplus {S}_{i+k}\right)\Theta {S}_{i}-f$$

The multi-scale difference top-hat enhancement operator is as follows:

12 $${f}_{e}=f+\left({f}_{t\left(i+k\right)} {\Theta g}-{f}_{b\left(i+k\right)}{\Theta g}\right)$$

where g is a 3 $\times$ 3 structural element.

13 $$g=\left[\begin{array}{ccc}0& 1& 0\\ 1& 1& 1\\ 0& 1& 0\end{array}\right]$$

**Figure 2:**
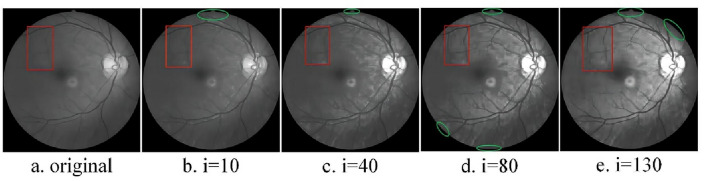
Differential multiscale morphological top-hat transformation.

**Figure 3:**
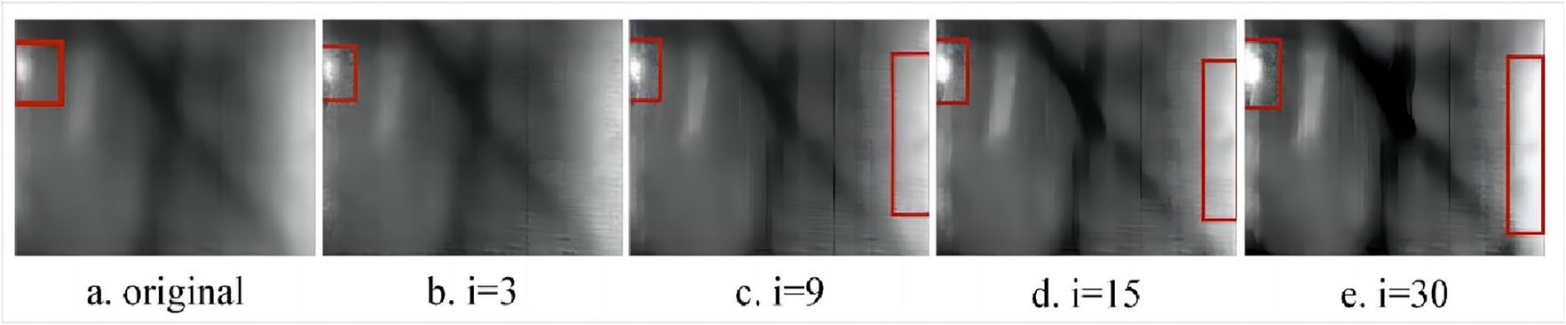
Conventional morphology multi-scale top-hat transformations.

**Figure 4:**
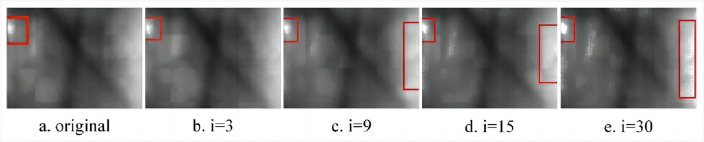
Differential multiscale morphological top-hat transformation.

Under the scale of structural elements (i = 10, 40, 80, 130, k = 10), the proposed new morphological top-hat transformation is applied to process the eyeball image, resulting in Fig. 2. A comparison between Fig. 1a–e and Fig. 2a–e reveals that increasing the scale of structural elements enhances the details within the region marked by the red rectangle. Notably, Fig. 1 exhibits a gradual onset of local over-enhancement in the area denoted by the green oval, whereas Fig. 2 maintains good stability at the same scale. Similarly, employing the same approach with scale structural elements (i = 3, 9, 15, 30, k = 15) yields Figs. 3 and 4. Examining Figs. 3a–e and 4a–e shows that escalating the scale of structural elements enhances the details within the area marked by the red rectangle on the left. A detailed comparison of the two images reveals significant disparities, including the gradual emergence of local over-enhancement in the marked areas on the left and right in Fig. 3. In contrast, Fig. 4 demonstrates superior robustness at the same scale. By visually contrasting the two sets of images, it is evident that the newly proposed morphological top-hat transformation in this article outperforms the traditional morphological top-hat transformation, showcasing enhanced efficacy in image enhancement.

### Adaptive iteration cutoff threshold

In the previous section, this paper has thoroughly discussed the different effects of traditional morphological operators and new morphological operators on image enhancement at multiple identical scales. The following content will focus on how to select the optimal scale to achieve the best image enhancement goal. For differential multi-scale morphological top-hat transformations, the choice of scale space is crucial and requires further optimization and research. In the field of one-dimensional signal processing, references^26–31^ have conducted in-depth studies on the ability of structural elements to extract signal characteristics at different scales. The results indicate that as the scale of the structural elements increases, their ability to extract signal features first improves and then declines. This finding provides an important reference for selecting the optimal scale in image enhancement in this paper.

**Figure 5:**
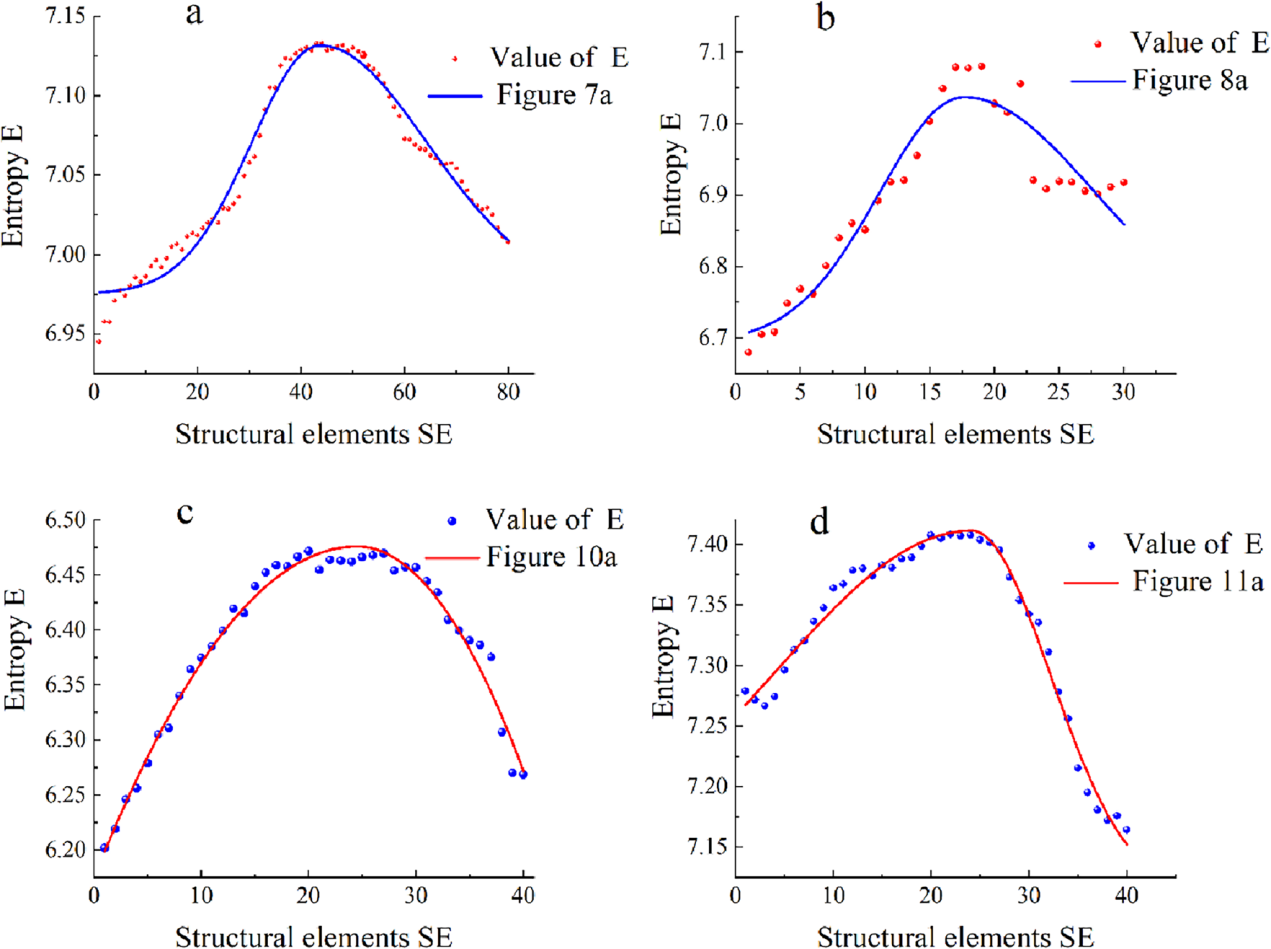
Variation of E extraction capacity with SE.

In the field of 2D image processing, Mello et al. explored the utilization of information entropy in multi-scale morphological image enhancement^32^. They derived an index, entropy (E), that signifies the image's feature extraction capability. This study involved the selection of two sets of laboratory-prepared defective infrared images and two sets of damaged thermal images of wind turbine blades as research subjects to validate the effectiveness and applicability of this index in the realm of infrared image enhancement research. The experimental findings are depicted in Fig. 5.

Figure 5a–d respectively represent the fitting curves of Figs. 7a, 8a, 10a, and 11a. The experimental results indicate that the trend of entropy changes similarly to the trend of signal feature extraction. With the increase of structural elements, the capability of extracting image features first increases and then decreases, with an optimal scale range existing. Therefore, this paper proposes an iterative thresholding method based on E to select a reasonable scale, using the maximum value of E in the image as the extremum of the objective function to determine the selected scale value i.

Entropy (E)^33^ in image processing is a measure that quantifies the information complexity within an image. It is determined by computing the probability of each pixel intensity value's occurrence based on the image's histogram distribution. The expression for entropy is given by:

14 $$E\left(f\right)=-{\sum }_{k=0}^{L-1}P\left(k\right){\text{log}}_{2}\left(P\left(k\right)\right)$$

where* f* is the original image, k is the value of pixels in the image, and P(k) is the probability of occurrence of value k in the image. If b is the number of bits for the image, then $\text{L}={2}^{b}$ (for grayscale images, b = 8). The higher the entropy, the richer the details and information content in the image, and vice versa.

In the experiments, it was found that determining the proportion of structural elements based only on the maximum value of E may lead to over-enhancement of the image. Therefore, we introduced a stopping criterion to control the number of iterations of the enhancement algorithm. At each step, the rate of change of entropy, EI, is calculated by comparing the absolute difference between the entropy of the previous step and that of the current image, and dividing this value by the absolute value of the entropy of the current image. The specific calculation formula is as follows:

15 $$\begin{array}{c}EI=\frac{\left|{E}_{i}-{E}_{i-1}\right|}{\left|{E}_{i}\right|}\end{array}$$

When EI is greater than a specific constant $\upzeta$, the iterative enhancement process is halted. In general, the stopping criterion is defined as:

16 $$\begin{array}{c}\frac{\mid {E}_{i}-{E}_{i-1}\mid }{E}>\zeta \end{array}$$

The selection of the constant ζ is influenced by two factors: it should be sufficiently small to ensure the convergence of the solution, and large enough to satisfy the requirements for the choice of structural element scale. The selection of ζ can refer to the stopping criterion for anisotropic diffusion defined by Sequeira et al.^34^.

### AICT-DMTH method steps

**Figure 6:**
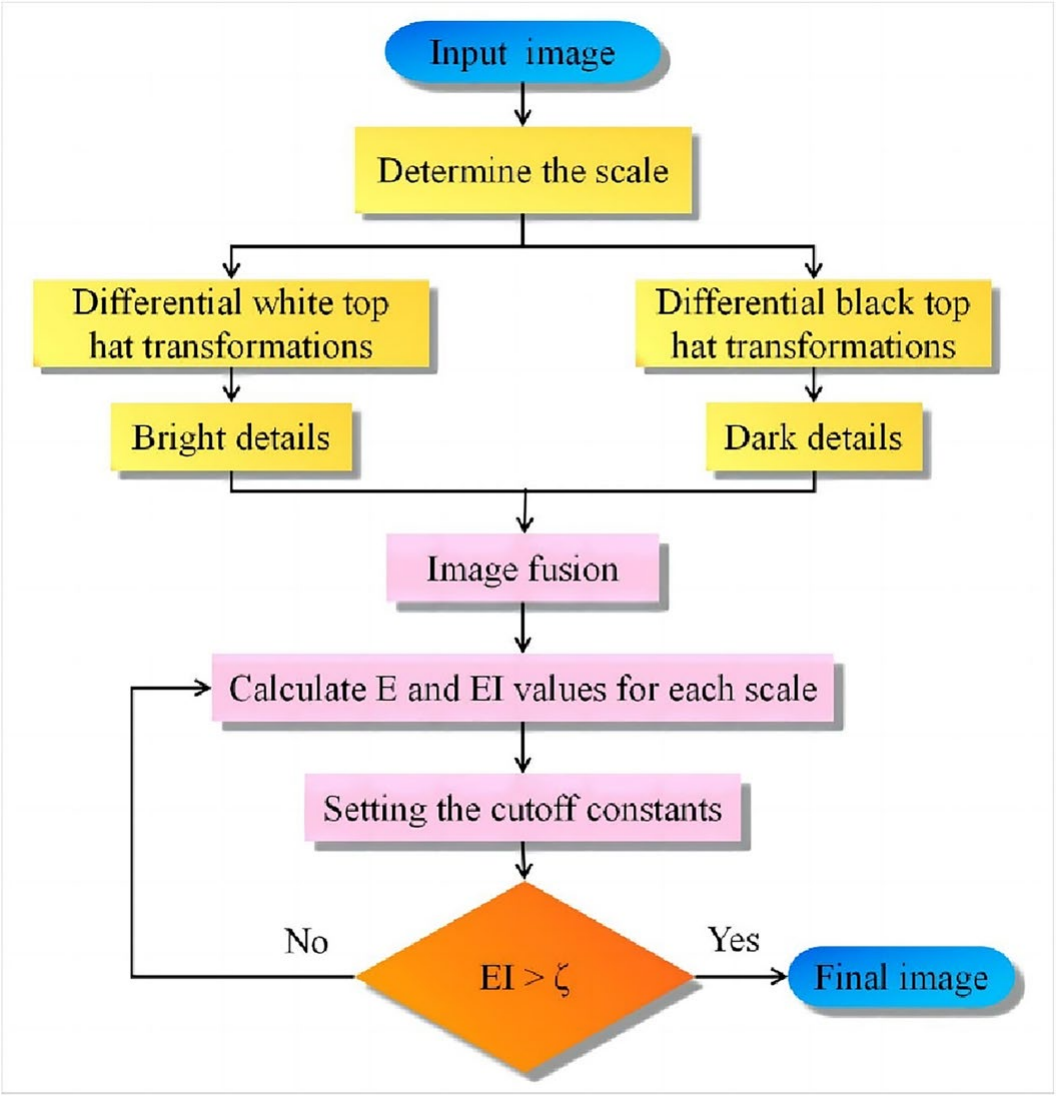
Method flowchart.

The method proposed in this paper uses the objective function E as the condition for iterative termination to obtain the optimal scale, thereby enhancing the image detail features. The specific workflow is shown in Fig. 6.

*Step 1*: Input a grayscale image with size M × N.

*Step 2*: Determine the scale range (min(M, N)), perform white top-hat difference multiscale transformation on *f*, erode *f* with a circular structuring element of scale i to obtain *f*_*i*_​. Then, dilate *f*_*i*_​ with a circular structuring element of scale i + k to obtain *f*_*o*_​, where the bright detail image obtained from the white top-hat transformation is *f − f*_*o*_​. Similarly, by performing black top-hat difference multiscale transformation on *f*, obtain the dark detail image *f*_*c​*_* − f*.

*Step 3*: Obtain the enhanced image by weighted fusion of the original image and the detail images. In this process, an iterative method is used to calculate the E value for each scale.

*Step 4*: While maintaining a constant difference scale *k* between erosion and dilation, the scale i of the structuring element is gradually changed, and the EI value for each scale is calculated. The average of these EI values is determined and denoted as EI_A_, and twice the value of EI_A_ is set as the cutoff constant. When the EI value for a particular scale exceeds this cutoff constant, that scale i is selected as the optimal scale. After determining the optimal scale i, it is kept unchanged, and the same method is used to select the value for the difference scale k. Since the difference scale k is generally smaller, the maximum EI value is chosen as the cutoff constant in this case.

*Step 5*: Enhance the original grayscale image based on the obtained cutoff scale using the new difference multiscale morphological top-hat operator. Through this process, the enhanced image is finally obtained.

## Experiments

This section will introduce the preparation process of two experimental devices and their specimens. Additionally, through qualitative and quantitative analysis, this paper compares the AICT-DMTH method with existing advanced technologies. Laboratory-prepared specimens and thermal images of damaged wind turbine blades from wind farms were selected as the research objects.

### Laboratory experiment

Two sets of advanced thermal excitation infrared non-destructive testing devices were specifically designed for different application scenarios. One set is used for sample testing in a laboratory environment, while the other is used for on-site testing of wind turbine blades. The indoor testing device includes two 1 kW halogen lamps, special heat insulation lampshades, an electronic control system, a small motor, an infrared thermal recorder (NECR300W2), a computer with an i5 processor, and image acquisition and processing software. To facilitate the study of infrared detection, experimental specimens with various defects were prefabricated. The specimens were made of unidirectional fiber cloth, laid in a cross pattern along the "0" and "90" directions, with a total of 6 layers and a thickness of 0.87 mm per layer. The defects included bubbles, cracks, and other imperfections.

**Figure 7:**
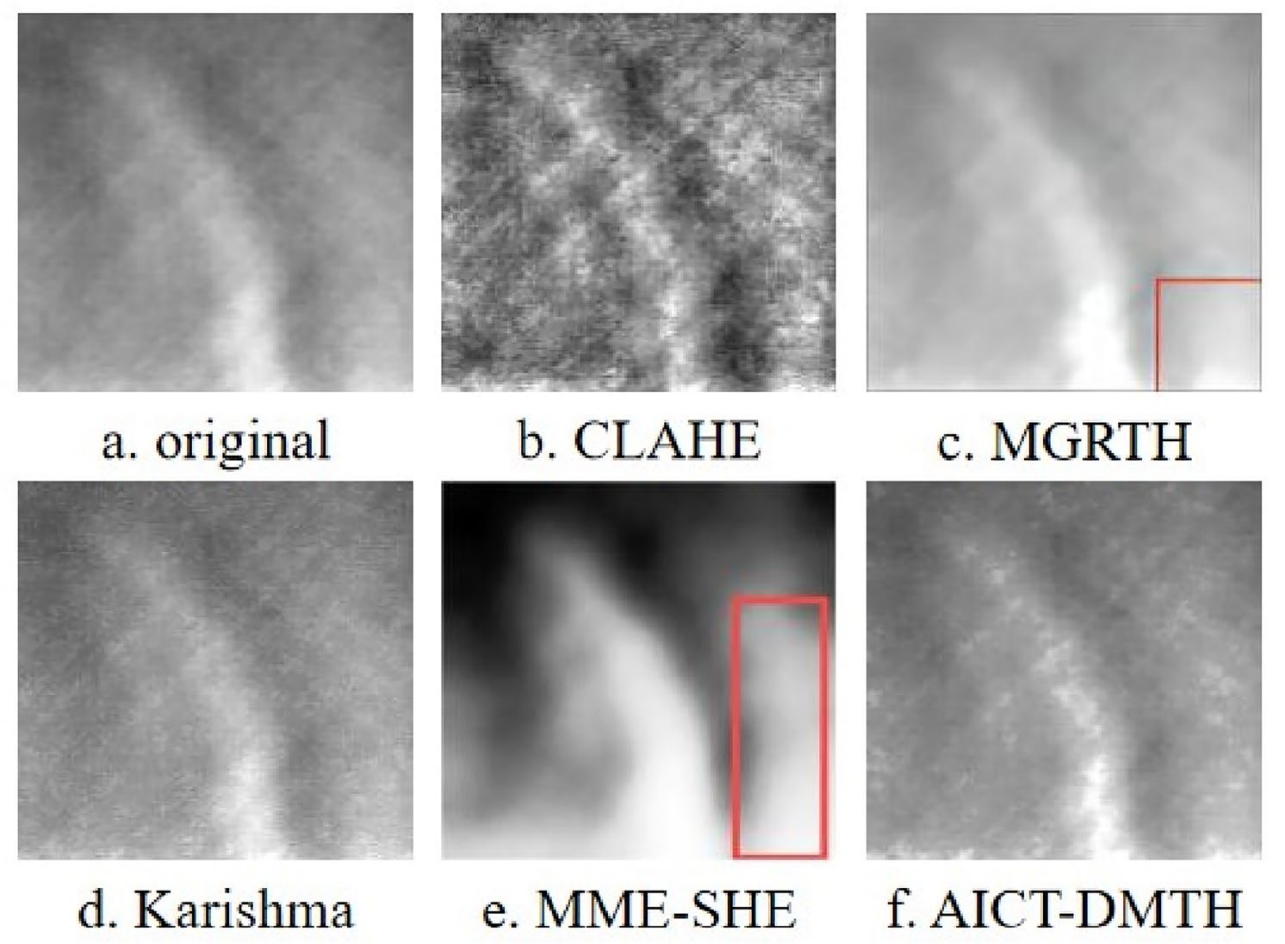
Infrared images of defective samples of prefabricated composites.

The infrared composite defect thermal image obtained from the experimental testing device is depicted in Fig. 7a. In order to validate the effectiveness of the proposed method, the thermal image in Fig. 7a underwent processing using the CLAHE, MGRTH, Karishma^35^, MME-SHE^42^ and AICT-DMTH methods, with the processing results shown in Fig. 7b–f.

The contrast of the image in Fig. 7b has been enhanced compared to Fig. 7a. However, this enhancement has resulted in increased noise, especially in regions of uniform brightness, leading to noticeable distortion. The overall brightness enhancement in Fig. 7c has led to over enhancement, as indicated by the red-marked region. While Fig. 7d shows some enhancement effects, they are not as pronounced. Figure 7e exhibits an over-enhancement of the contrast in the image's detail features, which results in severe edge blurriness and an increased background brightness within the area marked by the red box. In comparison to the four methods, Fig. 7f not only enhances the contrast but also improves the detail features of the image, making the defect edge characteristics clearer.

**Figure 8:**
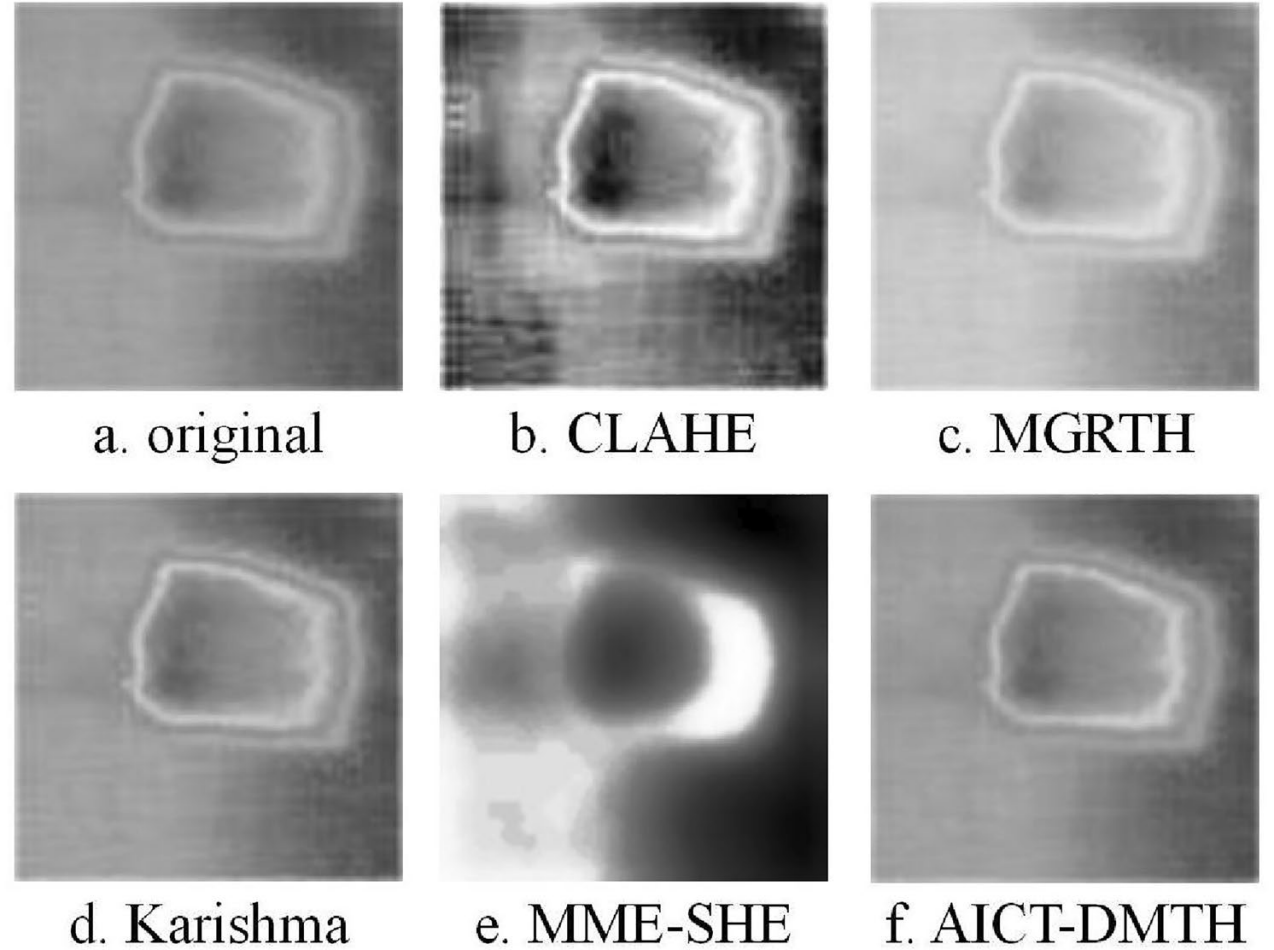
Infrared image of prefabricated bubble defective sample.

Figure 8a presents the thermal image of an infrared bubble defect obtained through an experimental testing apparatus. To validate the efficacy of the proposed method, the image in Fig. 8a was processed using CLAHE, MGRTH, Karishma, MME-SHE and our method, yielding Fig. 8b–f. Figure 8b exhibits severe distortion and the emergence of light pseudo-images. Figure 8c shows an overall increase in brightness but fails to enhance the details of the bubble defect edges. Figure 8d demonstrates some enhancement of the bubble edge features, yet the effect is not particularly pronounced. In Fig. 8e, the bottom right corner displays numerous optical artifacts, and because of the severe over-enhancement, the contours of the bubbles are no longer recognizable. Figure 8f displays a significant improvement in both contrast and detail, surpassing the original Fig. 8a.

### Wind power plant experiment

**Figure 9:**
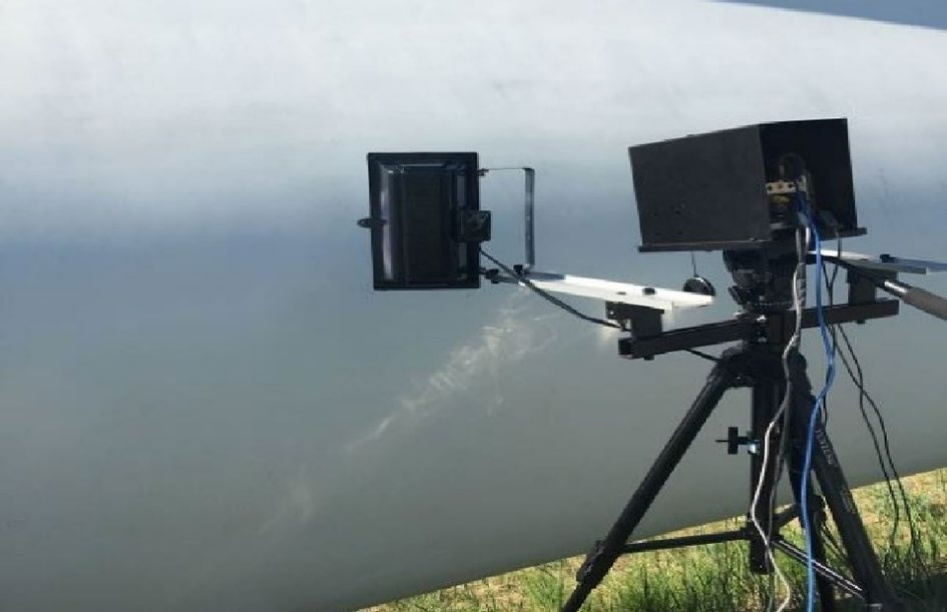
The outdoor test device.

In order to further validate the engineering applicability of the method, experimental research was conducted on the blades of a wind turbine at a wind farm in Inner Mongolia. The rated power of the wind turbine generator is 1520KW, the blade type is HI37, with a total length of 30 m and a maximum chord length of 3 m. The blade is mainly composed of a main beam and mounting plates. The main material of the main beam is FRP composite, and the main material of the mounting plates is PVC foam. Figure 9 illustrates the outdoor testing apparatus.

**Figure 10:**
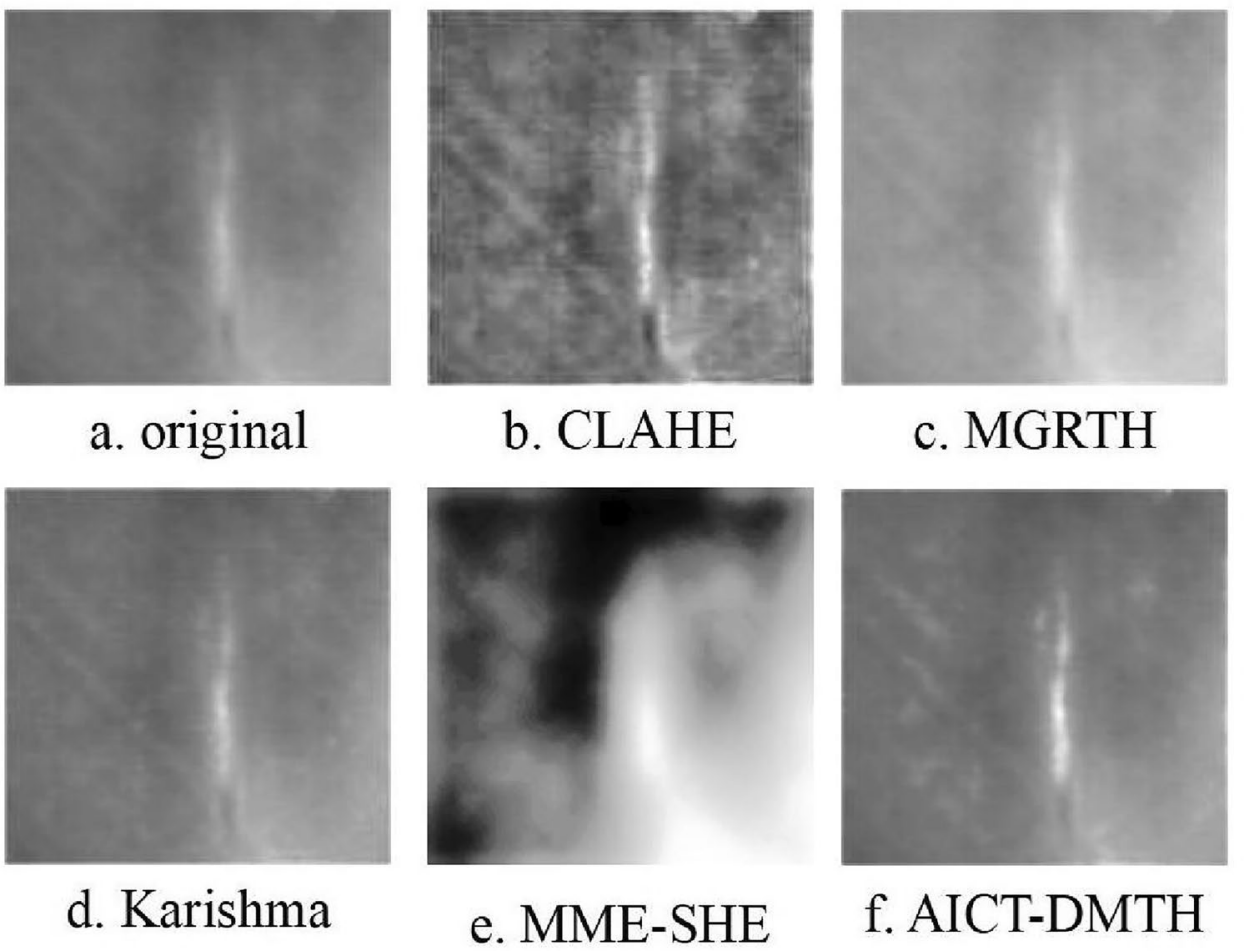
Infrared image of wind turbine blade crack defects.

**Figure 11:**
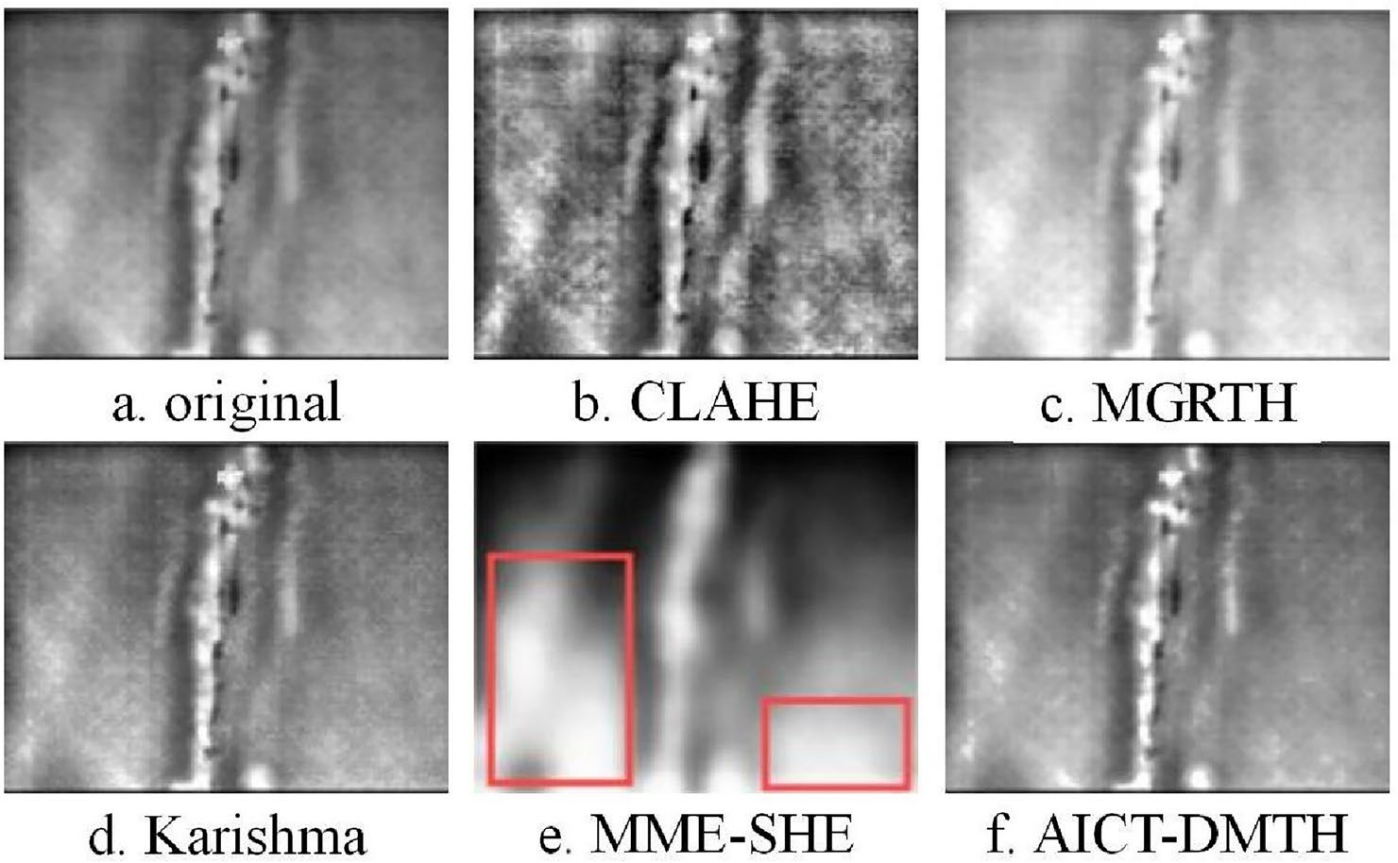
Infrared image of wind turbine blade crack defects.

**Figures 12:**
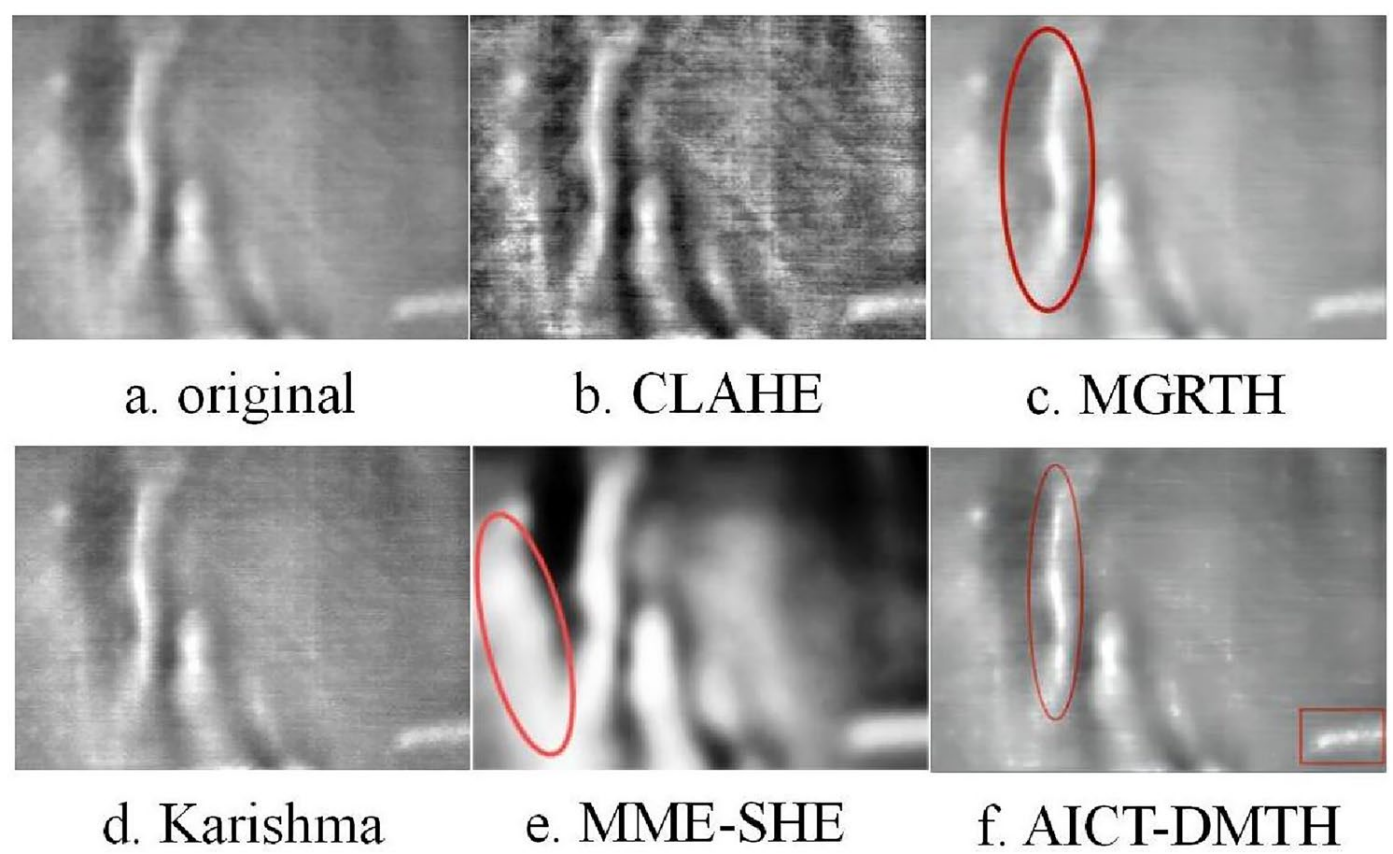
Infrared image of wind turbine blade folds.

Infrared defect thermal images of wind turbine blade main beams and Polyvinyl Chloride (PVC) were obtained using an infrared detection device, as shown in Figs. 10a, 11a, and 12a. These three images were processed using the CLAHE, MGRTH, Karishma, and AICT-DMTH methods, and the results are shown in Figs. 10, 11, and 12, respectively.

Figure 10a depicts a typical crack defect within wind turbine blades. Compared to Fig. 10a,b exhibits an increase in contrast; however, this comes at the expense of noise amplification in uniform areas, which results in the loss of fine details and clarity. In Fig. 10c, the edge characteristics appear blurred, the texture enhancement is subdued, and the contrast lacks definition. Figure 10d presents a more pronounced enhancement effect, yet it only intensifies the crack defect feature in areas with higher intermediate gray values relative to the original Fig. 10a, leading to the diminution of certain edge details. Because the cracks in the original Fig. 10a is small in size and have low contrast, the detail features of the image cannot be distinguished after enhancement with the MME-SHE algorithm. Ultimately, Fig. 10f preserves the original attributes while substantially sharpening the edge features and contrast, achieving an effective image enhancement outcome.

Figure 11a presents the infrared depiction of a crack defect within a wind turbine blade. In Fig. 11b and c, the contrast has been augmented relative to the original image in Fig. 11a; nonetheless, this enhancement compromises the distinctness of the edge features, leading to a more obscured appearance. In Fig. 11e, the background within the red rectangular box exhibits excessive enhancement, which after enhancement, has led to further blurring of the edges of the detail features and resulted in the loss of details. Figure 11d and f demonstrate superior enhancement effects. However, when compared to Fig. 11f, Fig. 11d exhibits somewhat diminished clarity in the edge features. Figure 11e attains image enhancement while preserving good stability.

Figure 12a depicts a common folding defect in wind turbine blades. Contrasting with Fig. 12a, Fig. 12b enhances the defect's contrast, yet the overall image is significantly disrupted by noise, resulting in notable distortion. In Fig. 12c, the defect area highlighted in red appears wider compared to the original image, potentially adversely affecting defect diagnosis. Figure 12d demonstrates a fairly effective enhancement. In Fig. 12e, the background within the red oval box is overly enhanced, and the detail features in the image have become blurry, which is not conducive to fault diagnosis. Figure 12f adeptly filters out noise while enhancing the defect features, rendering the edge details in the red-marked area more distinctly visible. This indicates that Fig. 12f excels in feature enhancement and noise filtration, thereby facilitating more precise defect diagnosis in the image.

### Results and discussion

To further showcase the superiority of the proposed method, the following metrics have been chosen as quantitative evaluation indicators to validate the proposed approach. The Peak Signal-to-Noise Ratio (PSNR) is utilized as an assessment criterion for image quality^36,37^, addressing the limitation of Mean Squared Error's strong reliance on image intensity. A higher PSNR signifies an improved processing effect on the image. The formula for PSNR is defined as follows:

17 $$\begin{array}{c}PSNR\left(f,{f}_{E}\right)=10\times lo{g}_{10}\frac{(L-1{)}^{2}}{MSE\left(f,{f}_{E}\right)}\end{array}$$

18 $$\begin{array}{c}MSE\left(f,{f}_{E}\right)=\frac{1}{M\times N}\sum_{u=0}^{M-1} \sum_{v=0}^{N-1}({f}_{E}\left(u,v\right)-f\left(u,v\right){)}^{2}\end{array}$$

where MSE is the mean square error, *f* is the original image, *f*_*E*_ is the processed image. L is the grey level of the image with size M $\times$ N.

Relative Enhancement in Contrast (REC)^38,39^, quantify the enhancement effect of image contrast. A larger REC value indicates a more significant improvement in image contrast. REC is defined as:

19 $$\begin{array}{c}REC=\frac{C\left({f}_{E}\right)}{C\left(f\right)}\end{array}$$

where *f* is the leaf infrared image, *f*_*E*_ is the enhanced image and C is the image contrast. C is defined as:

20 $$\begin{array}{c}C\left(f\right)=20\times \text{log}\left[\frac{1}{M\times N}\sum_{u=1}^{M} \sum_{v=1}^{N} \left((f(u,v){)}^{2}-(\frac{1}{M\times N}\sum_{u=1}^{M} \sum_{v=1}^{N} f(u,v){)}^{2}\right)\right]\end{array}$$

where M $\times$ N is the size of the image and (u, v) are the spatial coordinates.

Absolute Mean Brightness Error (AMBE)^40^, it quantifies the degree to which the average brightness of the enhanced image is preserved. A small AMBE value indicates that the enhanced image retains its average brightness. AMBE is defined as follows:

21 $$\begin{array}{c}AMBE\left(f,{f}_{E}\right)=\left|A\left(f\right)-A\left({f}_{E}\right)\right|\end{array}$$

where A(*f*) is the average brightness of the original image and A(*f*_*E*_) is the average brightness of the enhanced image.

Structural similarity (SSIM)^41^ is a metric between 0 and 1 used to evaluate the similarity between two images. A higher SSIM value indicates less difference between the two images, implying that the image enhancement method applied can better preserve the structural details of the image, resulting in improved visual effects. Its definition is:

22 $$\begin{array}{c}{\left.{\text{SSIM}}\left(x,y\right)=\left[\begin{array}{c}l\left(x,y\right)\end{array}\right.\right]}^{\alpha }{\left[\begin{array}{c}c\left(x,y\right)\end{array}\right]}^{\beta }{\left[\begin{array}{c}s\left(x,y\right)\end{array}\right]}^{\gamma }\end{array}$$

where $\alpha >0,\beta >0,\upgamma >0$, $l\left(x,y\right)$ is used for luminance comparison by computing the average brightness value of the image to describe its luminance characteristics. $c\left(x,y\right)$ is used for contrast comparison, employing standard deviation as a measure to assess the contrast level of the image. $s\left(x,y\right)$ is used for structure comparison, utilizing covariance as an indicator to evaluate the structural similarity of the image.

The numerical evaluation results of laboratory experiments and wind farm experiments are shown in the table below:

From the data presented in Table 1, it is evident that in the analysis results of the five sets of experiments, the PSNR values of the CLAHE, MGRTH and MME-SHE methods are relatively low, averaging 18.651, 16.796 and 13.853, respectively. In contrast, our method exhibits the highest PSNR value, averaging 36.209, significantly surpassing the other four methods. Specifically, compared to the Karishma method, which demonstrates superior enhancement effects, our method shows a 6.3% improvement in PSNR.

**Table 1:** Mean values of the indicators corresponding to the five methods.

Method	CLAHE	MGRTH	Karishma	MME-SHE	AICT-DMTH
PSNE	18.651	16.780	34.061	13.853	**36.209**
REC	0.914	**1.003**	0.984	0.774	0.984
AMBE	10.729	38.105	1.900	10.691	**0.940**
SSIM	0.636	0.964	0.905	0.603	**0.972**

The bold font indicates the optimal value for the corresponding metric.

The average REC values of the five methods are 0.914, 1.003, 0.984, 0.774 and 0.984, respectively. It can be seen that the proposed AICT-DMTH method has a higher REC value than the CLAHE and MME-SHE method. Compared to the MGRTH method, its REC value is slightly lower. This is because image enhancement involves a trade-off between noise and contrast, where better PSNR and AMBE values lead to poorer REC values. However, from a subjective evaluation perspective, our method's image enhancement is superior to the MGRTH method.

The AMBE values for the CLAHE, MGRTH and MME-SHE methods are notably high, indicating subpar performance in preserving average brightness during image enhancement. Notably, our proposed AICT-DMTH method has the smallest AMBE value, only 0.938. In contrast, the Karishma method has an AMBE value of 1.904, more than twice that of our method, highlighting the effectiveness of our method in maintaining the average brightness of the enhanced images.

Table 1 reveals that our method excels across multiple comparison metrics, particularly in the Structural Similarity Index (SSIM), a crucial performance indicator. SSIM plays a pivotal role in evaluating image quality by considering pixel intensity, contrast, and structural information, offering a comprehensive evaluation of visual perceptual quality. Thus, attaining the highest SSIM value indicates that our method better preserves image structural information, potentially delivering enhancement effects aligned with human visual characteristics. These quantitative analyses underscore the strong image enhancement capabilities and robustness of our method.

## Conclusions

This paper proposes an adaptive iterative cutoff threshold infrared image enhancement method based on a new differential multi-scale top-hat transform based on mathematical morphology. This method can effectively reduce the image instability caused by traditional multi-scale morphological top-hat transformation and eliminate the problem of feature size changes that may occur when the scale of the selected structural elements is too large. The proposed adaptive iterative threshold image weighting method can not only effectively enhance the details of infrared images, but also significantly improve the clarity of feature edges. This study selected 5 groups of infrared thermal images as experimental objects, and used CLAHE, MGRTH, Karishma, MME-SHE and AICT-DMTH methods for qualitative and quantitative verification respectively. Experimental results show that compared with the Karishma method with better enhancement effect, this method achieves 6.3% peak signal-to-noise ratio PSNR and 7.4% structural similarity SSIM improvement, and its absolute average brightness error AMBE is only half of the Karishma method. This further confirms the effectiveness and potential of this method in infrared image enhancement of leaves.

Infrared thermal wave technology plays a vital role in the detection of wind power blades. By capturing and analyzing the heat wave distribution on the blade surface, it can non-destructively pinpoint potential defects inside the blade and accurately assess their size and characteristics. Experimental studies have shown that this technology significantly improves the accuracy of defect identification and plays a key role in clearly defining defects within the blade. In addition, infrared thermal wave technology has laid a solid foundation for the subsequent application of deep learning for intelligent image classification, thus promoting the intelligent process of wind turbine blade maintenance and management.

## Data Availability

Figures 1 and 2 are from Ref.^25^. All other data supporting the main conclusions of this study can be found in the main text.
